# Limitations in diagnostics and quantification of small lesions with low uptake in the clinical context of prostate ^18^F/^68^Ga-PSMA PET/MRI

**DOI:** 10.1186/s40658-026-00902-3

**Published:** 2026-06-15

**Authors:** Maike E. Lindemann, Walter Jentzen, Alina Küper, Pedro Fragoso Costa, Marcel Gratz, Lale Umutlu, James Nagarajah, Stephan G. Nekolla, Ivo Rausch, Ken Herrmann, Harald H. Quick, David Kersting

**Affiliations:** 1 High-Field and Hybrid MR Imaging, University Hospital Essen, University of Duisburg-Essen, Essen, Germany; 2 Department of Nuclear Medicine, University Hospital Essen, University of Duisburg-Essen, Essen, Germany; 3 German Cancer Consortium (DKTK, Partner Site Essen), Essen, Germany; 4 Erwin L. Hahn Institute for Magnetic Resonance Imaging, University of Duisburg-Essen, Essen, Germany; 5 Department of Diagnostic and Interventional Radiology and Neuroradiology, University Hospital Essen, University of Duisburg-Essen, Essen, Germany; 6 Department of Radiology and Nuclear Medicine, Radboud University Medical Center, Nijmegen, The Netherlands; 7 Department of Nuclear Medicine, Klinikum Rechts Der Isar, School of Medicine and Health, Technical University of Munich, Munich, Germany; 8 QIMP Team, Center for Medical Physics and Biomedical Engineering, Medical University of Vienna, Vienna, Austria

**Keywords:** ^18^F-PSMA PET/MR, ^68^Ga-PSMA PET/MR, Radionuclide therapy planning, Prostate cancer, ^177^Lu-PSMA therapy

## Abstract

**Purpose:**

The aim of this study was to investigate the limits of diagnostics and therapy planning for patients with prostate cancer using non-time-of-flight ^18^F/^68^Ga-PSMA PET/MRI under clinically challenging imaging conditions with small lesion sizes and low uptake. Lesion detectability and quantification accuracy were evaluated for different acquisition and reconstruction parameters in a systematic phantom study and subsequent on patient data.

**Methods:**

PET/MRI measurements were performed using a small lesion NEMA phantom. PET data were acquired for nine different activity concentrations (AC). Data of a longer single-bed protocol in the pelvis or a shorter whole-body protocol were reconstructed using relative or absolute scatter correction (SC). PET images were analysed considering a ± 25% deviation range between imaged and true AC as acceptable. Thirteen PSMA-PET/MRI patients with primary lesions or lymph node metastasis < 12 mm in the pelvis were included in this study. The presence of the halo artefact was evaluated in six ^18^F-PSMA and seven ^68^Ga-PSMA PET/MRI patients. For 21 lesions (diameter 6.4–12.3 mm) in total, the AC was quantified.

**Results:**

For both radiotracers, the 9.7 mm sphere was still visible at 0.16 kBq/mL with emission times > 40 min. The 3.7 mm sphere was only detectable at 22 kBq/mL with emission times > 4 min. All spheres ≥ 6.5 mm provide acceptable quantification at an AC of 1.32 kBq/mL for ^18^F PET/MRI protocols of ≥ 12 min and 2.75 kBq/mL for ^68^Ga PET/MRI protocols. In phantom data, no halo artefact was observable and different SC methods had no impact on quantification. 4/6 ^18^F-PSMA patients and 7/7 ^68^Ga-PSMA patients showed a halo around the bladder using relative SC, which could be reduced in all patients using absolute SC. Comparing the minimum quantifiable AC (MQAC) from in the phantom study as a threshold to the patient data, all lesions provided acceptable quantification with values > MQAC (AC 3.3–108.5 kBq/mL) for equal reconstruction and acquisition parameters.

**Conclusions:**

The results demonstrated that the detection of lesions in the sub-centimetre range and a reliable quantification of ^18^F/^68^Ga-PSMA uptake using standard acquisition and reconstruction parameters within clinical PET/MRI protocols is possible. This allows for an individual assessment of potential therapy options for each patient.

## Introduction

Prostate cancer is one of the most frequent cancers in men worldwide with good survival when diagnosed in an early stage, but with poor prognostics in advanced metastasized disease stages [1, 2]. The prostate-specific membrane antigen (PSMA), a membrane protein with a remarkable overexpression in most prostate cancer cells, is established as an ideal target for either diagnostic imaging or radionuclide therapy of prostate cancer patients [3–7].

Over the last decade, ^18^F- and ^68^Ga-labelled PSMA positron emission tomography/magnetic resonance imaging (PET/MRI) became important for the detection and quantification of prostate cancer [5, 8–11]. PET/MRI is known to offer an excellent soft tissue contrast and reduced radiation exposure. The excellent soft tissue contrast and additional functional imaging parameters (e.g. diffusion-weighted imaging) leads to a superior ability of MRI compared computed tomography (CT) imaging to detect even small lymph node metastases in the sub-centimetre range and results in improved diagnostics of prostate cancer patients [12].

The assessment of potential therapy options for prostate cancer is an individual process for each patient and dependent on their age, constitution, disease stage and personal preferences. The current clinical practice to target smaller single prostate lesions is potentially radiotherapy if dissection is not applicable [13]. PSMA-PET-guided radiotherapy planning increasingly relies on accurate delineation of small PSMA-avid lesions. The accurate assessment of small lesion volumes provided by the superior soft tissue contrast of MRI may optimize target volume definition for radiation therapy [13, 14]. ^177^Lu-PSMA radionuclide therapy is approved in metastatic castration-resistant prostate cancer, where small lymph node and bone metastases can fall within the sub-centimetre range and may benefit from accurate pre-therapeutic dosimetry [16]. Emerging trials are also investigating ^177^Lu-PSMA in neoadjuvant and earlier disease settings [16–20]. Across both applications, accurate detection and quantification of small, low-uptake lesions on PSMA-PET/MRI is the foundational requirement. However, there are some well-known challenges with MR-based attenuation correction of PET data, which consequently limit the volume delineation and quantification of uptake [21–24]. In addition, a potential challenge for diagnostics and quantification of prostate cancer using PSMA PET/MRI is the so-called *halo artefact*, which is often caused by inaccurate scatter correction (SC) [25–27]. The halo artefact results in reduced signal intensity around the kidneys and bladder in PET images and lesions in this region might be non-detectable or uptake values are distorted and quantification is hampered.

Today, ^177^Lu PSMA radio-ligand therapy is increasingly applied to prostate cancer patients in advanced disease stages with large tumour volumes. However, it is anticipated that also prostate cancer patients in earlier disease stages with small tumour volumes (< 10 mm) may also benefit from this treatment option [7, 28, 29]. Those patients usually have a longer survival rate and thus, a careful assessment of therapy efficiency and toxicity in organs at risk are mandatory, which might be clarified by appropriate dosimetry based on pre-therapeutic acquired PET/MRI data.

The aim of this study was to investigate the limits of diagnostics and therapy planning for patients with prostate cancer using non-time-of-flight ^18^F/^68^Ga-PSMA PET/MRI under clinically challenging imaging conditions, such as small lesion sizes and low radiotracer uptake. Lesion detectability and quantification accuracy were evaluated for different PET/MRI acquisition and reconstruction parameters in a systematic phantom study and subsequent on patient data. This allows possible therapy options to be discussed individually for each patient.

## Materials and methods

### Small lesion phantom preparation

In this study, an abdominal NEMA phantom (Data Spectrum Corporation, Durham, USA) with six small glass spheres (wall thickness 0.7 ± 0.2 mm) was used to evaluate the lesion detectability and quantification performances of the radionuclides ^68^Ga and ^18^F [30]. The glass spheres had an inner sphere diameter of 9.7 mm, 8.9 mm, 7.7 mm, 6.5 mm, 4.8 mm and 3.7 mm [30]. The spheres and the large phantom cavity were filled with a radioactive solution of ^18^F-fluorodesoxyglucose or ^68^Ga. The ^68^Ga stock solution for the spheres and the large cavity contained acid to prevent adsorption on the phantom walls. The activity for both radionuclides was measured using a dose calibrator CRC-15R (Capintec Inc., Ramsey, USA).

For both radionuclides, an initial sphere activity concentration (AC) of approximately 22 kBq/mL and a sphere-to-background ratio of 20:1 were chosen to mimic clinical realistic imaging conditions for prostate cancer lesions [31, 32]. After the initial PET/MR measurement with a sphere AC of 22 kBq/mL, eight subsequent PET/MRI measurements were performed realising lower sphere ACs down to 0.08 kBq/mL. The time difference between each subsequent PET/MRI acquisition was approximately one half-life of ^18^F or ^68^Ga, respectively. Radionuclide dose calibrator-based (true) sphere ACs for all measurements are listed in Table 1. This phantom setup resembles typical lesion sizes and AC ranges of lymph node metastasis of prostate cancer patients under clinical challenging, but still realistic imaging conditions [31].

**Table 1:** Calculated sphere activity concentrations of all nine ^18^F and [^68^Ga] (within square parentheses) PET/MRI measurements are given as well as the results of the visual detectability analysis

Sphere activity concentration	Acquisition time	Sphere diameter					
		9.7 mm	8.9 mm	7.7 mm	6.5 mm	4.8 mm	3.7 mm
Visual detectability of ^18^F and [^68^Ga]							
21.50 kBq/mL [22.37 kBq/mL]	60 min	1 [1]	1 [1]	1 [1]	1 [1]	1 [1]	1 [1]
	40 min	1 [1]	1 [1]	1 [1]	1 [1]	1 [1]	1 [1]
	20 min	1 [1]	1 [1]	1 [1]	1 [1]	1 [1]	1 [1]
	12 min	1 [1]	1 [1]	1 [1]	1 [1]	1 [1]	1 [1]
	4 min	1 [1]	1 [1]	1 [1]	1 [1]	1 [1]	1 [0]
10.67 kBq/mL [11.16 kBq/mL]	60 min	1 [1]	1 [1]	1 [1]	1 [1]	1 [1]	1 [1]
	40 min	1 [1]	1 [1]	1 [1]	1 [1]	1 [1]	1 [1]
	20 min	1 [1]	1 [1]	1 [1]	1 [1]	1 [1]	1 [0]
	12 min	1 [1]	1 [1]	1 [1]	1 [1]	1 [0 (1)]	0 [0]
	4 min	1 [1]	1 [1]	1 [1]	1 [1]	0 [0]	0 [0]
5.33 kBq/mL [5.51 kBq/mL]	60 min	1 [1]	1 [1]	1 [1]	1 [1]	1 [1]	1 [1]
	40 min	1 [1]	1 [1]	1 [1]	1 [1]	1 [1]	1 [1]
	20 min	1 [1]	1 [1]	1 [1]	1 [1]	1 [1]	0 (1) [1]
	12 min	1 [1]	1 [1]	1 [1]	1 [1]	1 [0 (1)]	0 [0 (1)]
	4 min	1 [1]	1 [1]	1 [1]	1 [1]	1 [0]	0 [0]
2.64 kBq/mL [2.75 kBq/mL]	60 min	1 [1]	1 [1]	1 [1]	1 [1]	1 [1]	1 [0]
	40 min	1 [1]	1 [1]	1 [1]	1 [1]	1 [0 (1)]	1 [0]
	20 min	1 [1]	1 [1]	1 [1]	1 [1]	1 [1]	1 [0]
	12 min	1 [1]	1 [1]	1 [1]	1 [1]	0 [1]	0 [0]
	4 min	1 [1]	1 [1]	1 [1]	1 [0]	0 [0]	0 [0]
1.32 kBq/mL [1.36 kBq/mL]	60 min	1 [1]	1 [1]	1 [1]	1 [1]	1 [0]	0 [0]
	40 min	1 [1]	1 [1]	1 [1]	1 [1]	0 [0]	0 [0]
	20 min	1 [1]	1 [1]	1 [1]	1 [1]	0 [0]	0 [0]
	12 min	1 [1]	1 [1]	1 [1]	1 [1]	0 [0]	0 [0]
	4 min	1 [1]	1 [1]	0 [0]	0 [0]	0 [0]	0 [0]
0.66 kBq/mL [0.68 kBq/mL]	60 min	1 [1]	1 [1]	1 [1]	1 [1]	0 [0]	0 [0]
	40 min	1 [1]	1 [1]	1 [1]	1 [1]	0 [0]	0 [0]
	20 min	1 [1]	1 [1]	1 [1]	0 (1) [0]	0 [0]	0 [0]
	12 min	1 [1]	1 [1]	1 [1]	0 [0]	0 [0]	0 [0]
	4 min	0 [0]	0 [0]	0 [0]	0 [0]	0 [0]	0 [0]
0.33 kBq/mL [0.34 kBq/mL]	60 min	1 [1]	1 [1]	1 [0]	1 [0]	0 [0]	0 [0]
	40 min	1 [1]	1 [1]	1 [0]	0 [0]	0 [0]	0 [0]
	20 min	1 [1]	1 [0 (1)]	1 [0]	0 [0]	0 [0]	0 [0]
	12 min	1 [1]	1 [0]	0 (1) [0]	0 [0]	0 [0]	0 [0]
	4 min	0 [0]	0 [0]	0 [0]	0 [0]	0 [0]	0 [0]
0.16 kBq/mL [0.17 kBq/mL]	60 min	1 [1]	1 [0 (1)]	0 [0]	0 [0]	0 [0]	0 [0]
	40 min	1 [1]	1 [1]	0 [0]	0 [0]	0 [0]	0 [0]
	20 min	0 [0]	0 [0]	0 [0]	0 [0]	0 [0]	0 [0]
	12 min	0 [0]	0 [0]	0 [0]	0 [0]	0 [0]	0 [0]
	4 min	0 [0]	0 [0]	0 [0]	0 [0]	0 [0]	0 [0]
0.08 kBq/mL [0.08 kBq/mL]	60 min	0 [1 (0)]	0 [0]	0 [0]	0 [0]	0 [0]	0 [0]
	40 min	0 [0]	0 [0]	0 [0]	0 [0]	0 [0]	0 [0]
	20 min	0 [0]	0 [0]	0 [0]	0 [0]	0 [0]	0 [0]
	12 min	0 [0]	0 [0]	0 [0]	0 [0]	0 [0]	0 [0]
	4 min	0 [0]	0 [0]	0 [0]	0 [0]	0 [0]	0 [0]

Detectability was rated (1 = detected sphere, 0 = not detected sphere) on reconstructed PET images for different acquisition times and activity concentrations. Differences in detectability between relative and absolute (within round parentheses) scatter scaling are marked

### Image acquisition and reconstruction

All PET/MRI measurements in this study were performed on an integrated 3-Tesla whole-body PET/MRI system without time-of-flight modelling (Biograph mMR, Siemens Healthineers AG, Forchheim, Germany). For both measurement series (^18^F and ^68^Ga), the small lesion phantom was placed on a Styrofoam block centrally in the PET/MRI field-of-view. PET list-mode data was acquired for 60 min in one bed position per measurement. All nine PET/MRI measurements in each series with decreasing ACs in the phantom were taken in one go without repositioning of the phantom.

All PET reconstructions in this study were performed retrospectively using the e7-tools (Siemens Healthineers, Molecular Imaging, Knoxville, USA). According to a typical clinical PET/MRI protocol for prostate imaging, PET list-mode data was divided into different acquisition times. The longer time frames of 60 (reference measurement), 40 and 20 min (resembling a clinical focussed single-bed position measurement in the pelvis region) were reconstructed using the ordinary Poisson ordered-subsets expectation maximization (OP-OSEM) algorithm. The shorter 12 and 4 min time frames (resembling a clinical whole-body measurement) were reconstructed using OP-OSEM combined with point spread function (PSF) modelling. PSF modelling improves the image quality by reducing the image noise and therefore, PSF may be beneficial for lesion detection. However, PSF modelling is also known to cause edge artefacts and a quantitative image analysis may be hampered [33]. Due to the longer acquisition times in clinical focussed PET/MRI protocols (> 20 min), PET images provide a better count statistic and improved image quality by itself. For shorter acquisition times (< 12 min) in whole-body PET/MRI, it is beneficial to include PSF modelling to take advantage of less noisy PET images for lesion detection. For all reconstructions, 3 iterations, 21 subsets and a Gaussian post-filter with 4 mm full width at half maximum were used. In all PET reconstructions, a three-dimensional single Compton scatter simulation with either relative or absolute scaling of the estimated scatter was applied. The resulting PET images have matrix dimensions of 344 × 344 × 127 resulting in a voxel size of 2.09 × 2.09 × 2.03 mm^3^. For attenuation correction of the phantom, a registered CT-based attenuation correction map was used. The standard, vendor implemented prompt gamma correction for ^68^Ga was applied.

### Phantom image analysis

All PET reconstructions (including all slices in the PET data) were evaluated in a human observer study to assess the visual detectability of each sphere in the phantom under different acquisition and reconstruction parameters. The visual sphere detectability was rated by five nuclear medicine physicians on an established 3-point-scale (0 = not detectable, 1 = detectable, but comparable to noise and 2 = clearly detectable). A sphere was rated as detected if the sum score of all ratings was ≥ 5. PET reconstructions were evaluated anonymized and in random order.

The signal-to-noise ratio (SNR) was calculated in each sphere in all PET reconstructions. Therefore, six spherical volume-of-interests (VOIs) with corresponding diameter of the six spheres were manually positioned in identical planes and positions in all PET reconstructions using PMOD version 4.202 (PMOD Technologies Ltd, Zurich, Switzerland). The average AC in each sphere was measured ($\overline{{AC }_{sphere}}$). For the placement of the VOIs the co-registered attenuation correction map was used. To assess the AC in the background, 35 circular regions-of-interests with a diameter of 20 mm were placed in an axial slice in the phantom (same slice as the centres of the spherical VOIs) [30]. The mean background AC over all VOIs ($\overline{{AC }_{background}}$) and standard deviation (${\sigma}_{background}$) of AC_background_ was then used to calculate the SNR as follows (Eq. 1):

1 $$SNR=\frac{\left|\overline{{AC }_{sphere}}- \overline{{AC }_{background}}\right|}{{\sigma}_{background}}$$

To assess the lesion detectability under challenging imaging conditions, the size-dependent minimum detectable activity concentration (MDAC) was estimated. Therefore, a threshold SNR was defined to indicate visual sphere detectability using a histogram of the SNR distribution of detected or non-detected spheres. To determine the MDAC for each sphere, the SNR was analysed as a function of the AC for each sphere and each reconstruction. To reduce the influence of image noise, a cubic spline regression analysis was performed [30]. The MDAC was then calculated as the AC at the threshold SNR [30].

A quantification analysis for different ACs, PET acquisition times and SC methods was performed. Imaged ACs in the spheres can be biased due to the partial volume effect (PVE). For the correction of the PVE, a contour-based or an oversize-based method provide equally reliable results [32, 34]. For the contour-based PVE correction, recovery coefficients for each sphere were calculated from the 60 min PET acquisition. The average imaged AC within each sphere contour was then divided by the diameter-dependent recovery coefficient to calculate the corrected AC [34]. For the oversize-based PVE correction, an oversized VOI was placed around each sphere including the entire lesion activity. The oversized VOI contains contribution from the background activity and thus, a background subtraction was performed to obtain the true AC in the sphere [34]. The AC in the background was measured in a VOI placed close to the sphere. To evaluate the accuracy of quantification in each sphere, the ratio between PVE-corrected to true ACs was calculated. A deviation ratio range of ± 25% was regarded as acceptable under these challenging conditions.

### Patient image analysis

All patients signed informed consent and all patient measurements were performed in accordance with the Declaration of Helsinki and approved by the institutional ethics committee (University of Duisburg-Essen, medical faculty, Essen, ethics committee approval number: 11‐4822-4825-BO). For the patient evaluation, prostate cancer patients with small lymph node lesions and low uptake, comparable to the phantom setting in this study, were retrospectively included. The institutional image database was browsed for ^18^F-PSMA-1007 avid (June 2023–August 2025) or ^68^Ga-PSMA-11 avid (February 2019–June 2021) primary prostate cancer lesions or lymph node metastases in the pelvis. All patients with pelvic primary lesions or lymph node metastasis < 12 mm were evaluated for further analysis, but only 20 lesions with lower signal-to-background ratios were finally included in this study. Patients were referred to a clinical indicated PSMA PET/MRI because of either suspected primary prostate carcinoma (8/13 patients) or biochemical recurrence (5/13 patients). In total, six ^18^F-PSMA-1007 PET/MRI patients with eleven lesions (6 primary lesions, 5 lymph node metastasis) and seven ^68^Ga-PSMA-11 PET/MRI patients with ten lesions (7 primary lesions, 3 lymph node metastasis) were evaluated and compared to the corresponding phantom data.

The effective diameter of all lesions (13/21lesions) was determined manually from MRI images. Therefore, a diagnostic contrast-enhanced T1-weighted trans-axial VIBE MR sequence (volume interpolated breath-hold examination) was used with a pixel spacing of 0.78 × 0.78 mm^2^, a slice thickness of 3 mm, 1.49 ms echo time, 3.64 ms repetition time and phase encoding in anterior–posterior direction. If morphologically not discernible (8/21lesions), PET images were used to evaluate the effective lesion diameter with an iterative segmentation algorithm [35]. For the patient cohort, a median activity of 287 MBq (range 187 MBq—322 MBq) ^18^F-PSMA-1007 and respectively, 91 MBq (range 84–145 MBq) ^68^Ga-PSMA-11 was administrated. The clinical PET/MRI data were acquired on average 117 min (range 69–126 min) post ^18^F-PSMA-1007 administration and 79 min (range 65–110 min) post ^68^Ga-PSMA-11 administration. PET list-mode data was acquired for a 20 min or 40 min in a clinical focussed single-bed position measurement in the pelvis region for the ^18^F-PSMA-1007 patient cohort (except one 12 min whole-body examination) and for 12 min/bed in a whole-body examination (except one 20 min single-bed measurement) for the ^68^Ga-PSMA-11 patient cohort. PET image reconstruction was performed with identical parameters compared to the phantom image reconstruction. For attenuation correction of the PET patient data, a standard Dixon-VIBE-based segmentation approach with an additional bone atlas and HUGE truncation correction was used [36]. The presence of the halo artefact was visually rated (non, slight, moderate or strong) in each reconstruction. Contour-based PVE correction using recovery coefficients from the phantom study were applied as described for the phantom analysis, because most lesions were located close to the bladder (to avoid falsification of the results due to the influence of the bladder for the oversize approach). To compare phantom and patient data, the minimum quantifiable activity concentration (MQAC) derived from the phantom setup was used to assess if the lesion uptake in patients is reliable for given acquisition and reconstruction parameters. Lesion ACs > MQAC indicates acceptable quantification even in small or low uptake lesions.

### Absorbed dose estimations

The primary aim of this study was to evaluate lesion detectability and quantification accuracy of PSMA-PET/MRI for different acquisition and reconstruction parameters in a systematic phantom study and subsequent on patient data under clinical challenging imaging conditions with small lesion sizes and low radiotracer uptake. This allows for an individual assessment of potential therapy options for each patient dependent on their age, constitution and disease stage. One potential therapy option is the ^177^Lu-PSMA-617 radionuclide therapy. In this context, the question arises if smaller lesions with low uptake potentially respond to radionuclide therapy, which could further improve patient selection and optimizes treatment options for prostate cancer patients. Therefore, the therapeutic absorbed dose of ^177^Lu-PSMA-617 using a simple dosimetry model was calculated for phantom and patient data.

For the phantom measurements, the MDAC was used to predict the absorbed dose. For each MDAC, the therapeutic absorbed dose of ^177^Lu-PSMA was calculated using a simple dosimetry model, which is described more in detail below, to investigate the clinical relevance of radionuclide therapy in non-detectable lesions. According to the clinical PET/MRI protocol, a median applied activity of 287 MBq ^18^F-PSMA and respectively, 91 MBq ^68^Ga-PSMA was assumed. PET/MRI data acquisition started typically 117 min post injection for ^18^F-PSMA and respectively, 79 min post injection for ^68^Ga-PSMA imaging. To assess the absorbed dose response for each lesion a threshold of > 10 Gy was used after an administration of 7.4 Gy ^177^Lu-PSMA in a first cycle of treatment [37, 38].

For each lesion in the patient data, the therapeutic absorbed dose of ^177^Lu-PSMA-617 using a simple dosimetry model was calculated based on the pre-therapy PET data to investigate the clinical relevance of radionuclide therapy planning in the context of PSMA PET/MRI of patients with small lesions with low uptake. The PET uptake values for each lesion were calculated using the PVE-corrected AC, the lesion volume and the administrated activity. For the absorbed dose estimation, a correction with regard to differences in physical half-lives of diagnostic radiotracers ^18^F (*T*_*F*_), respectively ^68^Ga (*T*_*Ga*_), and the therapeutic radiotracer ^177^Lu (*T*_*Lu*_) was mandatory. To predict the ^177^Lu-PSMA-617 uptake value *U*_*Lu*_*(t*_*PET*_*)* from the determined ^18^F-PSMA-1007 uptake value *U*_*F*_*(t*_*PET*_*)*, respectively ^68^Ga-PSMA-11 uptake value *U*_*Ga*_*(t*_*PET*_*)*, following Eq. 2 was used (exemplarily for ^18^F) [28]:

2 $${U}_{Lu}\left({t}_{PET}\right)={U}_{F}\left({t}_{PET}\right)*{e}^{\frac{\mathit{ln}\left(2\right)}{{T}_{F}}{t}_{PET}}*{e}^{-\frac{ln(2)}{{T}_{Lu}}{t}_{PET}}$$

This projected uptake value was used to extrapolate individual uptake curves, from which the ^177^Lu-PSMA-617 residence times or time-integrated activity coefficients (TIAC) were estimated (Eq. 3). For a simple approximation, we assumed all patients have the same mono-exponential decay with an effective half-live (*T*_*eff*_), which was determined by Peters et al. [28] with a mean *T*_*eff*_ of 82 h for tumour tissue.

3 $$TIAC={U}_{Lu}\left({t}_{PET}\right)*{e}^{\frac{\mathit{ln}\left(2\right)}{{T}_{eff}}{t}_{PET}}*\frac{{T}_{eff}}{ln(2)}$$

For each lesion, the projected TIAC and the mass were then used to predict the absorbed dose *D*_*mean*_ per unit administrated ^177^Lu-PSMA-617 activity using the sphere model in OLINDA/EXM 2.2 (Hermes Medical Solutions). Note that this is only a rough estimation of the absorbed doses. However, this prediction may give a first impression on clinical relevance of ^177^Lu-PSMA-617 therapy in small lesions with low uptake. To give an impression of the errors in dose estimation based on biological variation, the predicted absorbed doses were calculated for the mean ± standard deviation (SD) range (82 h ± 25 h) of the effective half-life from ^177^Lu-PSMA for each patient.To assess the absorbed dose response for each lesion a threshold of > 10 Gy was used to identify responders (any kind of PSA response) and non-responders after an administration of 7.4 Gy ^177^Lu-PSMA in a first fraction of treatment [37, 38].

## Results

### Minimum detectable activity concentration in phantom measurements

**Fig. 1:**
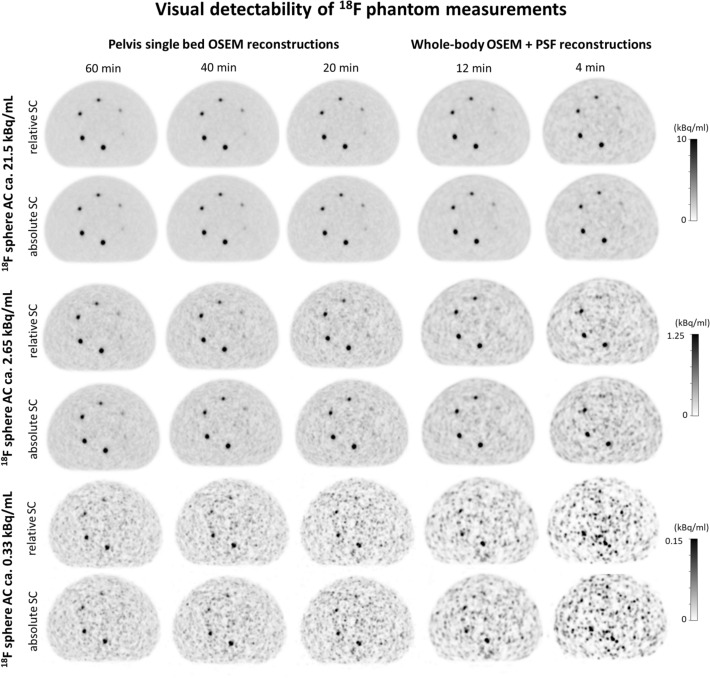
Exemplary PET images of ^18^F for high, medium and low activity concentration (AC, top to bottom) at five PET acquisition times (left to right) using relative or absolute scatter correction (SC) to evaluate the sphere detectability in the phantom measurements

**Fig. 2:**
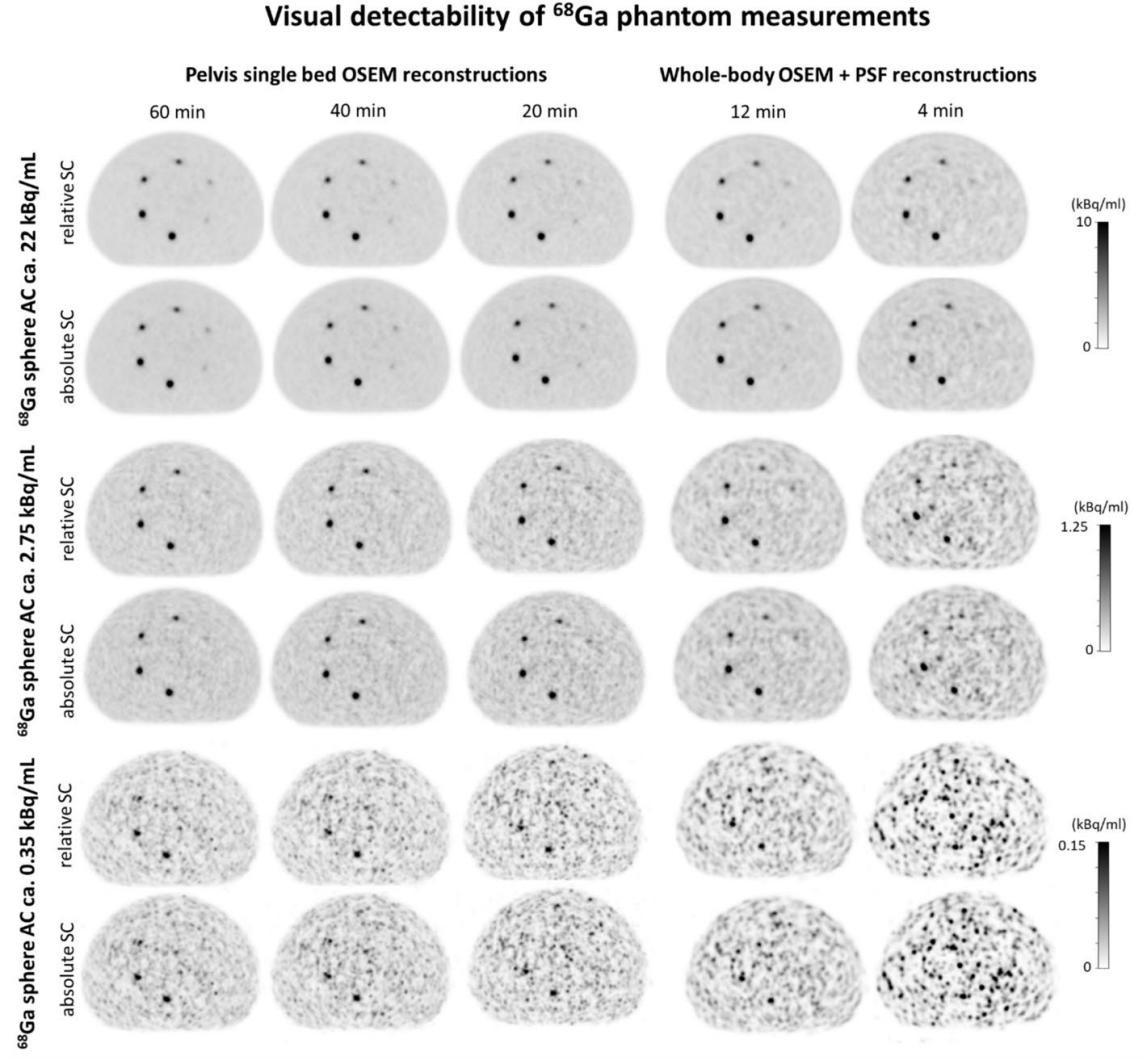
Exemplary PET images of ^68^Ga for high, medium and low activity concentration (AC, top to bottom) at five PET acquisition times (left to right) using relative or absolute scatter correction (SC) to evaluate the sphere detectability in the phantom measurements

Table 1 summarizes the results from visual detectability rating. Longer PET acquisition times and higher sphere ACs resulted in overall improved lesion detectability. There were no remarkable differences in detectability between absolute and relative scatter scaling in this analysis, even if absolute SC resulted in increased sphere visibility in single reconstructions (3/90 in ^18^F PET reconstructions and 6/90 in ^68^Ga PET reconstructions). These observations are visually supported by the exemplary PET images of the phantom in Figs. 1 and 2. Overall, ^18^F provided slightly increased sphere detection, especially for lower ACs and smaller spheres, compared to ^68^Ga in this phantom study. Higher ^18^F recoveries might be explained by smaller positron ranges. For ^18^F PET data, all spheres for all acquisition times are visible in the measurement with highest AC of 21.5 kBq/mL (Table 1, Fig. 1) and no sphere could be detected in the measurement with lowest AC of 0.08 kBq/mL (Table 1, images in Fig. 1 not shown). The two smallest spheres (3.7 and 4.8 mm) could not be detected in the shorter whole-body PET/MR measurements (12 and 4 min), but were still visible in the longer single-bed measurement (> 20 min) at an AC of 2.65 kBq/mL (Table 1, Fig. 1). In the ^18^F reconstructions, the two largest spheres (9.7 and 8.9 mm) could not be detected in the 4 min acquisition with a sphere AC of 0.66 kBq/mL, but are still detectable in the longer 40 and 60 min measurements with a sphere AC of 0.16 kBq/mL (Table 1). For the ^68^Ga PET reconstructions, all spheres for all acquisition times (except the shortest 4 min acquisition) are visible in the measurement with highest AC of 22.37 kBq/mL (Table 1, Fig. 2) and no sphere could be detected in the measurement with lowest AC of 0.08 kBq/mL (Table 1, images in Fig. 2 not shown). The two smallest spheres (3.7 and 4.8 mm) could not be detected in the shorter whole-body PET/MR measurements (12 and 4 min), but were still visible in the longer single-bed measurement (40 and 60 min) at an AC of 11.16 kBq/mL with relative SC (Table 1). In the ^68^Ga PET data, the two largest spheres (9.7 and 8.9 mm) could not be detected in the 4 min acquisition with a sphere AC of 0.68 kBq/mL, but are still detectable in the longer 40 and 60 min measurements with a sphere AC of 0.17 kBq/mL (Table 1).

**Fig. 3:**
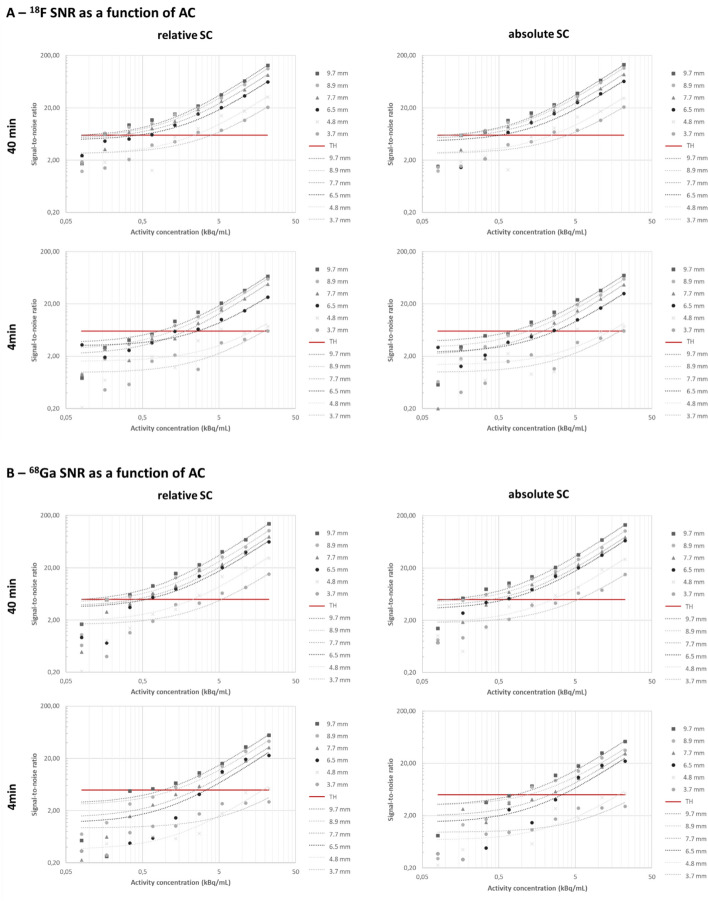
The signal-to-noise ratio (SNR) as a function of the activity concentration (AC) for ^18^F (**A**) and ^68^Ga (**B**) for all spheres and separately for both scatter correction methods (SC) are shown. The SNR is exemplary presented for a longer 40 min PET measurement (resembling a clinical focussed single-bed examination in the pelvis) and a shorter 4 min PET measurement (resembling a clinical whole-body examination). The red line indicates the threshold SNR for visual detectability. Dotted lines are fits from the regression for each sphere

Figure 3 shows the SNR as a function of AC for ^18^F (A) and ^68^Ga (B) exemplarily for a longer 40 min single-bed position and a shorter 4 min whole-body examination. For both radiotracers, the SNR increased with larger sphere diameter, with longer acquisition times and with higher ACs. No remarkable differences in SNR between relative and absolute SC was found. In a histogram analysis of all detected and not detected spheres, a SNR threshold value of SNR ≥ 6 for ^18^F and SNR ≥ 5 for ^68^Ga reconstructions was calculated. A SNR value larger than the threshold SNR value indicates visual detectability of a sphere. The MDAC for each sphere was obtained based on the derived threshold SNR values and are listed in Table 2. For both radiotracers, the MDAC decreased with longer PET acquisition times and with increasing sphere size. No remarkable deviations between relative and absolute SC were calculated. The results from the human observer study (Table 1) and the calculated MDACs are in good agreement, except for the largest 9.7 mm sphere. Here, the MDAC for both radiotracers indicated that the 9.7 mm should be visible in the 60 min reconstruction even at a sphere AC of 0.08 kBq/mL (Table 2, marked with an *x*), but this was not the case in the visual detectability analysis (Table 1). Overall, the 60 and 40 min PET reconstructions revealed comparable diagnostic information and MDACs for all spheres and both radiotracers. Note that PSF-reconstructed PET images are known for relatively low image noise levels and thus, increased SNR. Therefore, the longer 20 min OSEM and the shorter 12 min OSEM + PSF reconstructed images showed comparable MDACs (Table 2) and nearly equal visibility (Table 1) for the three larger spheres (9.7–7.7 mm) for both radiotracers.

**Table 2:** Calculated minimum detectable activity concentrations (MDAC in kBq/mL) for ^18^F (A) and ^68^Ga (B) for each sphere size, PET acquisition time and scatter correction (SC) method are shown

Sphere diameter (mm)	Relative SC					Absolute SC				
	60 min	40 min	20 min	12 min	4 min	60 min	40 min	20 min	12 min	4 min
*A – *^*18*^*F MDAC*										
9.7	x	0.08	0.20	0.20	0.81	x	0.10	0.19	0.19	0.73
8.9	0.11	0.12	0.25	0.25	1.21	0.10	0.12	0.20	0.23	1.18
7.7	0.26	0.23	0.31	0.61	1.79	0.22	0.24	0.32	0.25	1.84
6.5	0.31	0.43	1.28	0.96	2.58	0.25	0.50	0.95	1.03	2.68
4.8	1.32	2.39	2.79	4.98	13.63	1.26	2.45	2.80	5.27	13.96
3.7	2.52	3.94	7.30	13.59	19.18	2.56	3.94	6.49	13.19	18.89
*B – *^*68*^*Ga MDAC*										
9.7	0.08	0.17	0.25	0.93	x	0.11	0.20	0.21	0.81	0.08
8.9	0.17	0.34	0.38	1.30	0.15	0.18	0.28	0.37	1.18	0.17
7.7	0.41	0.61	0.67	2.51	0.37	0.40	0.60	0.51	2.33	0.41
6.5	0.56	0.78	0.97	3.41	0.51	0.60	0.91	0.95	3.32	0.56
4.8	2.28	2.77	8.24	18.66	2.07	2.49	2.86	7.44	18.32	2.28
3.7	5.44	8.05	14.85	x	4.94	5.40	8.84	14.01	x	5.44

Missing values (“x”) indicate that the calculated MDAC was outside the activity concentration range in this evaluation (< 0.08 kBq/mL or > 22 kBq/mL)

### Absorbed dose estimations in phantom measurements

The projected absorbed dose estimates of ^177^Lu-PSMA in each sphere based on the calculated MDACs (Table 2) for one cycle of 7.4 GBq are given in Table 3. Considering a threshold dose of > 10 Gy for any degree of response, the predicted absorbed doses imply that non-detectable lesions using the current implementation of the ^18^F-PSMA PET/MRI protocol (approximately 290 MBq administrated activity, start of measurement approximately 120 min post injection) did not respond to ^177^Lu-PSMA therapy. Even the smallest 3.7 mm sphere imaged with the shortest 4 min whole-body PET/MRI protocol showed a predicted absorbed dose < 10 Gy. For the current implementation of the ^68^Ga-PSMA PET/MRI protocol (approximately 90 MBq administrated activity, start of measurement approximately 80 min post injection), the predicted absorbed doses indicate that missed out lesions ≥ 6.5 mm were not clinically relevant for therapy assessment, because all those lesions showed a predicted absorbed dose < 10 Gy. Smaller lesions (4.8 mm and 3.7 mm), which are non-detectable in whole-body PET/MRI with shorter emission times (12 min or 4 min) might however respond to ^177^Lu-PSMA treatment (estimated absorbed dose > 10 Gy). With longer acquisition times in clinically focussed single-bed ^68^Ga-PSMA PET/MRI protocols (> 20 min), non-detectable lesions ≤ 4.8 mm might also not respond to ^177^Lu-PSMA therapy.

**Table 3:** The minimum detectable activity concentrations (MDAC) for ^18^F (A) and ^68^Ga (B) for each sphere size, PET acquisition time and scatter correction (SC) method were used to calculate the predicted absorbed dose (in Gy) of ^177^Lu-PSMA for one fraction of 7.4 GBq, given in this table

Sphere diameter (mm)	Relative SC					Absolute SC				
	60 min	40 min	20 min	12 min	4 min	60 min	40 min	20 min	12 min	4 min
*A – *^*18*^*F MDAC used to predict absorbed dose of *^*177*^*Lu at 7.4 GBq*										
9.7	x	0.04	0.11	0.11	0.43	x	0.05	0.10	0.10	0.39
8.9	0.06	0.06	0.13	0.13	0.64	0.05	0.06	0.11	0.12	0.62
7.7	0.14	0.12	0.16	0.32	0.94	0.12	0.13	0.17	0.13	0.97
6.5	0.16	0.22	0.67	0.50	1.35	0.13	0.26	0.50	0.54	1.40
4.8	0.68	1.24	1.45	2.58	7.06	0.65	1.27	1.45	2.73	7.23
3.7	1.30	2.02	3.75	6.98	9.86	1.32	2.02	3.34	6.78	9.71
*B – *^*68*^*Ga MDAC used to predict absorbed dose of *^*177*^*Lu at 7.4 GBq*										
9.7	x	0.14	0.30	0.45	1.66	x	0.20	0.36	0.37	1.45
8.9	0.14	0.30	0.61	0.68	2.31	0.27	0.32	0.50	0.66	2.10
7.7	0.69	0.73	1.08	1.19	4.45	0.66	0.71	1.06	0.90	4.13
6.5	0.90	0.99	1.38	1.71	6.01	0.90	1.06	1.60	1.68	5.85
4.8	3.44	3.98	4.84	14.39	32.6	3.62	4.35	5.00	13.00	32.00
3.7	9.26	9.43	13.95	25.74	x	8.56	9.36	15.32	24.28	x

Missing values (“x”) indicate that the calculated MDAC ranged outside the activity concentration range in this evaluation (< 0.08 kBq/mL or > 22 kBq/mL) and no absorbed dose could be calculated

### Minimum quantifiable activity concentration in phantom measurements

**Fig. 4:**
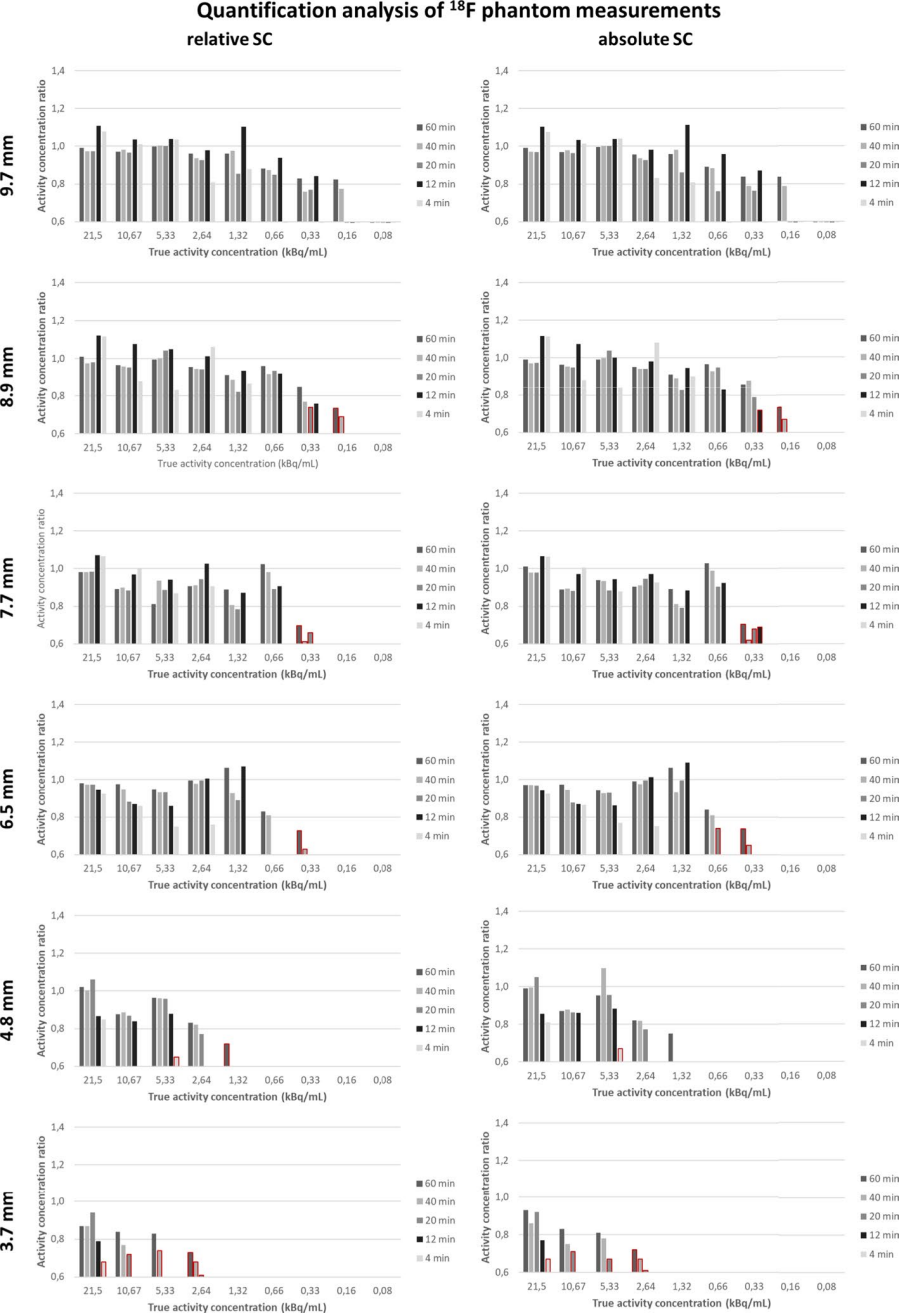
PET quantification performance analysis for ^18^F for all detected spheres (top to bottom) in this study are given for different activity concentrations (ACs), acquisition times and scatter correction (SC) methods (left to right). Ratios of partial volume effect corrected (oversize-based approach) to true ACs are presented. A deviation ratio range of ± 25% was regarded as acceptable under these challenging imaging conditions. Results that revealed noticeable deviations from the acceptance range are marked in red

**Fig. 5:**
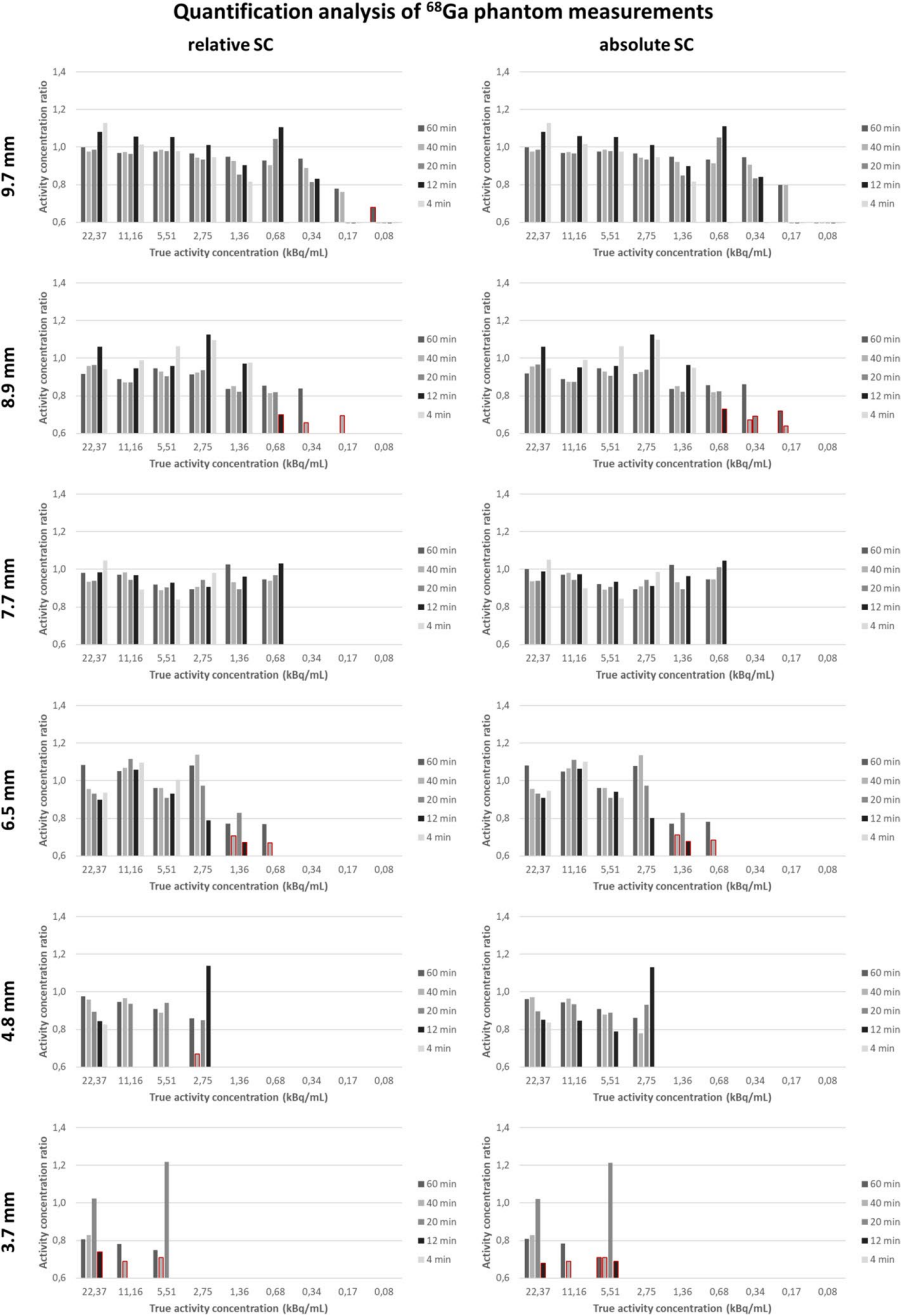
PET quantification performance analysis for ^68^Ga for all detected spheres (top to bottom) in this study are given for different activity concentrations (ACs), acquisition times and scatter correction (SC) methods (left to right). Ratios of partial volume effect corrected (oversize-based approach) to true ACs are presented. A deviation ratio range of ± 25% was regarded as acceptable under these challenging imaging conditions. Results that revealed noticeable deviations from the acceptance range are marked in red

Figures 4 and 5 summarize the quantification performance for the ^18^F and ^68^Ga PET/MRI phantom measurements at different acquisition times, sphere ACs and SC methods. Ratios of oversize-based PVE-corrected to true ACs are presented for all detectable spheres in this analysis. No systematic differences between OSEM or OSEM + PSF reconstruction algorithms, shorter or longer PET acquisition times and relative or absolute SC were observed. The quantification performance for both radiotracers exhibited a general trend towards a slight underestimation of PVE-corrected ACs (ratios < 1 in Figs. 4 and 5), especially for lower ACs. If the largest 9.7 mm sphere was detectable, the quantification performance was also acceptable within reasonable accuracy, which applied for both radiotracers. An accurate quantification of ACs in the smallest 3.7 mm sphere was only feasible in ^18^F and ^68^Ga PET/MRI measurements with highest ACs (> 21.5 kBq/mL) and longer PET acquisition times. For the 6.5 mm sphere, quantification was acceptable for ACs approximately above 1.3 kBq/mL for ^18^F and 2.75 kBq/mL for ^68^Ga for the longer single-bed PET/MRI measurements (> 20 min). For the shorter 4 min whole-body PET/MRI measurement, accurate quantification of the 6.5 mm sphere was possible approximately above 2.65 kBq/mL for ^18^F and 5.5 kBq/mL for ^68^Ga.

### Diagnostics, quantification and therapy response in patient measurements

The characteristics and quantitative analysis of eleven ^18^F-PSMA-1007 and ten ^68^Ga-PSMA-11 avid lesions are shown in Table 4. The effective diameter of all lesions ranged between 6.4 and 12.3 mm with an AC of 1.02 kBq/mL up to 108.28 kBq/mL and thus, the diameters fall within the scope of lesions in the sub-centimetre range and/or low PET uptake.

**Table 4:** Characteristics of eleven ^18^F-PSMA-1007 (A) and ten ^68^Ga-PSMA-11 (B) avid lesions are given.

Patient	Lesion	PET Tacq (min)	d_eff_ (mm)	RC	S/B ratio	Relative SC		Absolute SC		absSC/relSC AC ratio	Predicted absorbed dose of ^177^Lu-PSMA-617	
						Halo presence	PVE-corrected AC (kBq/mL)	Halo presence	PVE-corrected AC (kBq/mL)		TX Dose (Gy/GBq)	TX D_mean_ [SD range] (Gy) at 7.4 GBq
*A—*^*18*^*F-PSMA-1007 patient analysis*												
1	1	20	7.4	0.30	14.6	Slight	18.92	Non	21.99	1.162	1.29	9.53 [6.67—12.40]
	2	20	6.7	0.26	7.2	Slight	8.82	Non	11.17	1.267	0.65	4.83 [3.38 – 6.28]
2	3*	20	7.0	0.27	31.8	Nn	80.85	Non	81.82	1.012	3.80	28.13 [19.63 – 36.62]
3	4*	60	7.3	0.29	40.4	Moderate	91.31	Slight	92.89	1.017	7.36	54.47 [38.16 – 70.79]
	5	60	8.2	0.34	10.9	Moderate	11.84	Slight	17.51	1.479	1.39	10.31 [7.22 – 13.39]
	6	60	9.1	0.39	6.7	Moderate	11.28	Slight	14.44	1.280	1.15	8.52 [5.97 – 11–07]
4	7*	12	8.2	0.34	108.5	Non	108.28	Non	108.50	1.002	12.08	89.38 [62.60 – 116.18]
5	8*	40	8.4	0.35	11.2	Slight	11.39	Non	11.72	1.029	0.92	6.83 [4.79 – 8.88]
	9	40	8.2	0.34	7.7	Slight	6.44	Non	7.12	1.105	0.56	4.15 [2.91 – 5.39]
6	10	40	12.3	0.58	11.8	Moderate	16.36	Slight	17.41	1.064	1.17	8.68 [6.08 – 11.29]
	11*	40	7.1	0.28	28.0	Moderate	31.91	Slight	32.18	1.009	2.13	15.78 [11.05 – 20.51]
*B—*^*68*^*Ga-PSMA-11 patient analysis*												
1	1	12	7.1	0.23	45.3	Strong	22.71	Slight	25.39	1.118	6.13	45.38 [31.70 – 59.07]
	2	12	9.8	0.36	37.0	Strong	17.97	Slight	20.73	1.109	5.06	37.42 [26.14 – 48.70]
2	3*	12	10.0	0.37	11.0	Strong	3.06	Slight	3.30	1.079	0.79	5.84 [4.08 – 7.60]
3	4*	12	8.5	0.30	55.9	Moderate	28.16	Non	29.08	1.033	6.05	44.74 [31.23 – 58.26]
4	5	12	6.8	0.22	39.5	Slight	11.37	Non	11.86	1.043	2.95	21.86 [15.30 – 28.43]
5	6	20	6.4	0.20	16.5	Slight	6.06	Non	7.26	1.197	0.96	7.09 [4.95 – 9.24]
	7	20	12.2	0.48	9.7	Slight	5.31	Non	6.28	1.184	0.85	6.26 [4.37 – 8.16]
6	8	12	8.9	0.32	29.0	Strong	5.21	Non	8.49	1.630	1.85	13.70 [9.58 – 17.83]
	9	12	10.7	0.41	15.9	Strong	4.41	Non	7.34	1.666	1.61	11.92 [8.33 – 15.50]
7	10	20	11.7	0.46	18.0	Strong	1.02	Slight	1.62	1.589	0.46	3.42 [2.39 – 4.45]

Marked (*) lesions were located outside the halo artefact.

Recovery coefficients (RC) were used to correct the partial-volume-effect (PVE) of the imaged activity concentrations (AC). The signal-to-background (S/B) ratio is the PVE-corrected AC using absolute scatter correction (SC) divided by the background AC. The predicted absorbed dose of ^177^Lu-PSMA-617 for therapy (TX) based on the PET uptake in each lesion was calculated per administrated GBq and for one fraction of 7.4 GBq

**Fig. 6:**
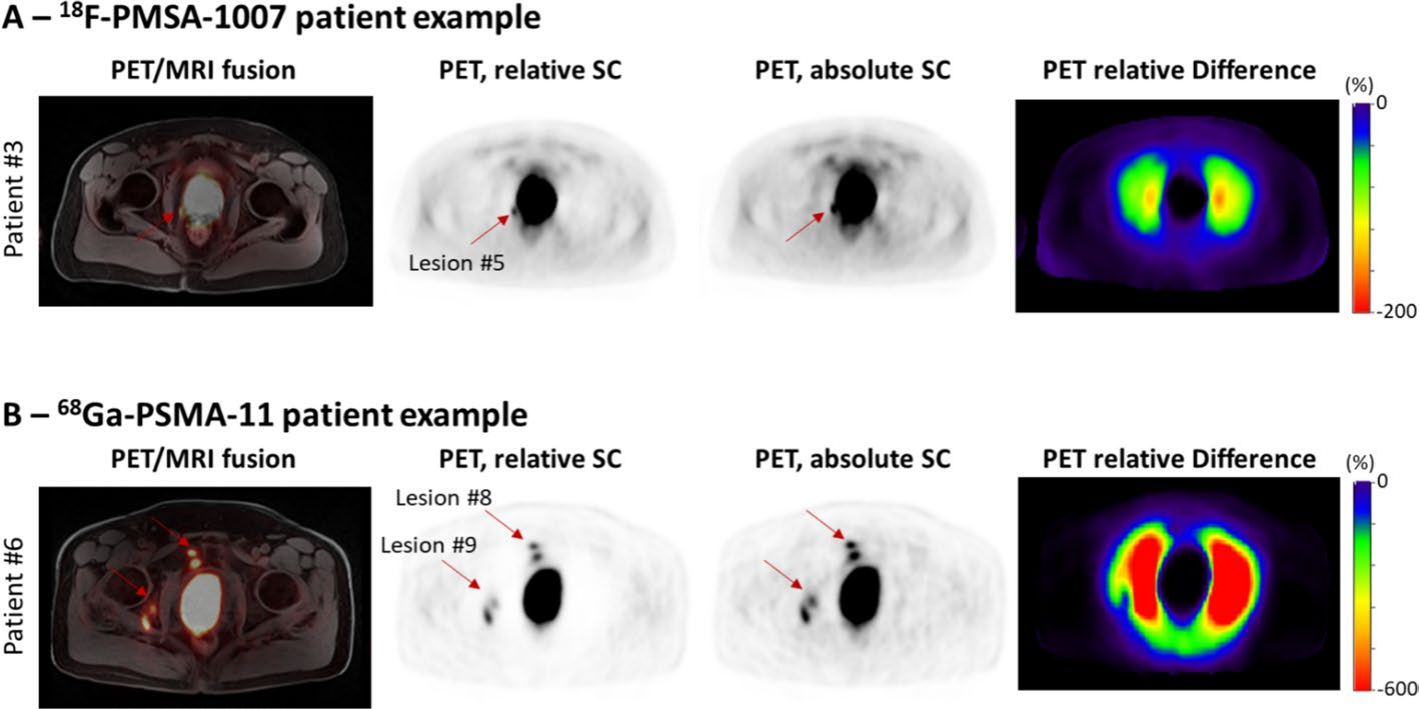
PET/MRI fusion images and transversal PET images, reconstructed using either scatter correction (SC) with relative or absolute scatter scaling, and relative difference images between PET reconstructions of a ^18^F-PSMA-1007 (**A**) and a ^68^Ga-PSMA-11 (**B**) PET/MRI patient example are shown. The halo artefact in the PET images with relative SC limits diagnostics and quantification of the marked lesion close to the bladder in both patient examples, but the presence of the artefact could be reduced using absolute SC. Relative differences in activity concentrations > -50% in the lesions were calculated

The rating of the halo presence in each reconstruction revealed that 4/6 ^18^F-PSMA-1007 patients and 7/7 ^68^Ga-PMSA-11 patients showed a halo around the bladder using relative SC. In all patients, the presence of the halo artefact could be reduced using absolute SC. In 2/7 ^18^F-PSMA-1007 patients and 3/7 ^68^Ga-PSMA-11 patients a slight presence of residual artefact is still visible. These patients have a body-mass-index of > 30 kg/m^2^. Lesion detection was not affected by the halo artefact in this study, but quantification was clearly biased (Table 4, Fig. 6). For lesions outside the halo artefact (marked with *), relative and absolute SC provide nearly equal AC, which is comparable to the results in the phantom study. The PVE-corrected AC is remarkably underestimated for lesions inside the artefact region using relative SC (Table 4, Fig. 6). The impact of SC depends on the relative location of the lesion relative to the halo artefact.

Compared to the calculated MQACs in each sphere in the phantom study (Figs. 4 and 5), all PVE-corrected lesion AC (absolute SC) in this patient study provided acceptable quantification (lesion ACs > MQAC) for given lesion size, AC and PET acquisition time and thus, could be used for therapy planning (Table 4). Note that the quantification of lesions #5, 6 and 10 (^18^F-PSMA-1007) and lesions #1, 2 and 10 (^68^Ga-PSMA-11) may be biased because of the residual halo artefact. The projected absorbed dose estimates in each lesion using 7.4 GBq ^177^Lu-PSMA-617 showed that 5/11 ^18^F-PSMA-1007 avid lesions and 6/10 ^68^Ga-PSMA-11 avid lesions might respond to radionuclide therapy considering a threshold first strike average dose of > 10 Gy for any degree of response. This implies that for lesion sizes in the sub-centimetre range and/or low radiotracer uptake patients could benefit from radionuclide therapy with ^177^Lu-labelled PSMA-617.

## Discussion

The phantom study results demonstrated that the detection of lesions in the sub-centimetre range and a reliable quantification of ^18^F/^68^Ga-PSMA uptake using standard acquisition and reconstruction parameters within typical clinical PET/MRI protocols is possible even under challenging imaging conditions with small lesion sizes and low uptake. Therefore, it indicates that PET/MRI allows to perform pre-therapeutic lesion dosimetry for an individualized therapy plan in patients with prostate cancer. However, the quantification performance revealed a general trend towards a slight underestimation in ACs. This underestimation has already reported in other publications and may be prone to low count statistics in the investigated lesion size and activity concentration ranges [32, 39, 40]. The inactive glass wall of the spheres may have an influence on the background spill-in effects, and consequently might impact our quantitative results. The spill-over effect especially impacts the calculated recovery coefficients used for contour-based PVE correction [41, 42]. However, this effect is not relevant for the oversize-based PVE correction, because the enclosed total activity is determined and corrected for background contribution. Both PVE correction approaches revealed comparable results with < 10% deviation in spheres ≥ 6.5 mm and < 15% for the 4.8 mm lesion. The overall good agreement between contour- and oversize-based PVE correction was supported by Kersting et al. and Hammersen et al. using the same phantom [32, 34]. Thus, the thin glass walls and the induced spill-over effects had only a limited impact on the quantification analysis in this study. PET images had a nearly cubic voxel size of 2 mm side length. The VOI for 4.8 mm sphere in the phantom measurements thus, contains approximately eight voxels. This small number of voxels is likely to cause deviations in the calculation of activity concentrations, especially for the contour-based PVE correction approach. The number of voxels increase when using the oversize-based PVE correction approach for the same sphere applying an oversize VOI with a diameter increment of two PET spatial resolutions and therefore, provide more robust quantification of activity concentration even for smaller sphere sizes [34]. However, in patient measurements we used the contour-based PVE correction, because most lesions were located close to the bladder, which may cause inaccuracies using the oversize PVE correction. The smallest lesion diameter in the patient study was 6.4 mm, which ended up in approximately 20 voxels in the VOI. In our opinion, this amount of voxel should provide a stable quantification of activity concentration even with the contour-based PVE approach.

PET imaging quality and quantification performance is dependent on the used PET system, reconstruction parameters and acquisition protocols. Therefore, the conclusions drawn in the manuscript are valid only for the chosen system and settings. Especially with the advent of a newer generations of PET systems including technical improvements in the field of PET detector technology, quantification limitations of small lesions will probably decrease. For example, the new-generation silicon-photomultiplier-based “digital” PET systems outperform the conventional PET systems in detector sensitivity and thus, improve lesion detection and quantification of uptake [43, 44]. The technical hardware improvements also enable software advances, such as time-of-flight reconstruction with very narrow time kernels [44], optimized PSF reconstructions [45], positron range correction for improved spatial resolution [46] or maximum-a-posteriori reconstructions [47] to allow for increased convergence. In this context, limits in lesion detection and quantification may be query. However, those improvements in PET imaging are not fully implemented for PET/MRI hybrid imaging yet and systematic performance measurements are mandatory anyways.

A potential limitation of this study is the transferability of an ideal phantom setup compared to individual patient data. In contrast to the phantom measurements, lesions may have a non-spherical geometry, an inhomogeneous uptake or their signal-to-background ratio may vary. Especially the latter impacts the recovery coefficients and therefore, contour-based PVE correction may cause deviation in the quantitative analysis in patient data compared to the fixed signal-to-background ratio in the phantom measurements. The level of background activity concentration is also important for lesion detection, reflected rather by the signal-to-background ratios than the lesion uptakes. Reduced sensitivity at off-centre positions may affect quantification accuracy and, in worth case, lesion detectability in patients with lesions in the periphery of the PET field-of-view. This may be even more pronounced in smaller lesions with low uptake. In clinical PET imaging usually overlapping bed acquisitions are used to correct for reduced sensitivity at bed edges [48]. Another constrain in patient data is that lesion ACs may be influenced by motion (patient motion, respiratory or cardiac motion). There are also differences in attenuation correction when comparing phantom and patient data. The phantom attenuation correction map is based on CT data, while the patient data was corrected using an MR-based attenuation correction map including a bone atlas and HUGE truncation correction [36]. MR-based attenuation correction has some well-known drawbacks compared to CT-based attenuation correction [23]. However, the latest versions of MR-based attenuation correction maps provide equivalent good results in quantification compared to CT-based attenuation corrected PET images (excluding the lung area) [49]. Thus, further clinical investigations are warranted to assess the impact of pre-therapeutic lesion dosimetry and individualized therapy planning using PET/MRI on therapy outcome in patients with prostate cancer.

The halo artefact occurs also in PET/CT imaging, but particularly in PET/MRI and is a challenge in daily routine, which hampers diagnostics and quantification of lesions inside the artefact region [26]. The halo artefact results from extreme differences in activity concentrations between the bladder/kidneys and surrounding background tissue, especially in ^68^Ga-PSMA PET imaging. The choice of SC method impacts the presence of the halo artifact in patients. However, in phantom measurements we used moderate sphere-to-background ratios, which are not likely to induce a halo. Thus, the different SC methods had no impact on lesion visibility and quantification accuracy in our phantom measurement. The most commonly used SC method in clinical PET imaging is based on the single-scatter simulation algorithm. Our study and previously results showed that using absolute scatter scaling reduces the presence of the halo artefact in nearly every patient and thus, improve diagnostics and quantification [27]. However, there are single patients with a residual halo artefact in the PET images corrected with absolute SC. These patients tend to have an increased body-mass-index, which is associated with overall large body dimensions causing more scattered events in general. Furthermore, patients with larger body dimensions may also exceed the conventional MR field-of-view not only with the arms positioned next to the body in PET/MRI, but also in the abdominal region. This may lead to truncation artefacts in the attenuation correction map and a bias estimation of scattered events [50]. The use of double-scatter simulation or multi-scatter methods, as well as the inclusion of an additive offset factor into the scatter model may lead to a reduction of the halo artefact in PET/MRI [51, 52].

Predicting the absorbed therapy dose based on pre-therapy measurements could help to select patients and optimize treatment for each patient individually by maximizing the absorbed dose in lesions while retaining the threshold absorbed doses for the organs at risk. Using a single time point ^18^F/^68^Ga-PSMA PET measurement to estimate the absorbed dose of ^177^Lu-PSMA therapy in lesions is feasible, but only to a limited extent [28]. First of all, the shape of the uptake time-activity curve is determined by the radiotracer kinetics, but radiotracer kinetics are highly variable in lesions, which potentially arises from heterogeneity in lesion biology. Thus, the estimation of absorbed doses based on general uptake pattern for lesions may result in remarkable differences inter-patient variability [28]. Moreover, we assumed that the different radiotracers used for diagnostics (^18^F-PSMA-1007 or ^68^Ga-PSMA-11) or therapy (^177^Lu-PSMA-617) have similar kinetics to project the absorbed dose of ^177^Lu-PSMA-617 based on PET data. It has been shown that the ligands indeed exhibit similar kinetic behaviour, but there are remaining differences between the PSMA ligands, which may influence absorbed dose estimation in target regions or organs at risk [53]. Our predicted absorbed dose of ^177^Lu-PSMA in lesions based on PSMA PET imaging is consequently just a rough approximation. Another general constrain is the lack of implementation of dosimetry in clinical routine, despite the evidence and benefit for an optimized treatment plan for each patient [54]. The 10 Gy threshold for any kind of treatment respond was a first assumption, but this absorbed dose threshold may be remarkably larger for patients with highly aggressive disease [37, 38]. Identifying responders and non-responders based on threshold doses needs a further expand in research.

While ^177^Lu-PSMA radionuclide therapy is currently established for advanced disease stages such as metastatic castration-resistant prostate cancer [16], accurate quantification of small and low-uptake lesions remains relevant within this population, where lymph node and bone metastases frequently fall in the sub-centimeter range. Moreover, for patients with isolated sub-centimeter lesions in earlier disease stages, radiation therapy belongs to standard of care and PET-guided planning can improve its outcome [14, 15]. The question addressed in this study, whether PET/MRI provides reliable quantification under these challenging imaging conditions, is of relevance to both applications, and becomes even more important, when ^177^Lu-PSMA therapy is investigated more frequently at earlier disease stages in current clinical trials [16, 19, 20, 28]. However, the study results showed that small lesion sizes and low uptake lesions potentially respond to radionuclide therapy with ^177^Lu-labelled PSMA. This may improve patient selection and optimizes treatment options for prostate cancer patients in earlier disease stages with small tumour volumes.

## Conclusion

This study aimed to systematically assess the boundaries of diagnostic performance and radionuclide therapy planning for prostate cancer patients when using non–time-of-flight ^18^F/^68^Ga-PSMA PET/MRI in demanding clinical imaging scenarios with small lesion sizes and low radiotracer uptake. The results showed that a reliable quantification of ^18^F/^68^Ga-PSMA uptake with PET/MRI is feasible and those lesions potentially respond to radionuclide therapy with ^177^Lu-labelled PSMA. PET imaging performance is dependent on the PET system itself, reconstruction parameters and acquisition protocol. Therefore, the conclusions drawn in the manuscript are valid only for the chosen system and settings. However, in this study it has been shown that PSMA PET/MRI could potentially be used to perform pre-therapy lesion dosimetry and individualized therapy planning in patients with prostate cancer.

## Data Availability

The datasets generated and/or analyzed during the current study are not publicly available due to privacy legislation but may be made available to qualified researchers on reasonable request from the corresponding author.
